# Supplementary material for: Fail-Safe System against Potential Tumorigenicity after Transplantation of iPSC Derivatives
Source: Stem Cell Reports. 2017 Mar 2;8(3):673–84. doi: 10.1016/j.stemcr.2017.02.003 (PMC5355810; doi:10.1016/j.stemcr.2017.02.003)
Supplement: Document S1. Figure S1 [file mmc1.pdf]

Stem Cell Reports, Volume *8*

Supplemental Information

Fail-Safe System against Potential Tumorigenicity after Transplanta-tion of iPSC Derivatives

Go Itakura, Soya Kawabata, Miki Ando, Yuichiro Nishiyama, Keiko Sugai, Masahiro Ozaki, Tsuyoshi Iida, Toshiki Ookubo, Kota Kojima, Rei Kashiwagi, Kaori Yasutake, Hiromitsu Nakauchi, Hiroyuki Miyoshi, Narihito Nagoshi, Jun Kohyama, Akio Iwanami, Morio Matsumoto, Masaya Nakamura, and Hideyuki Okano

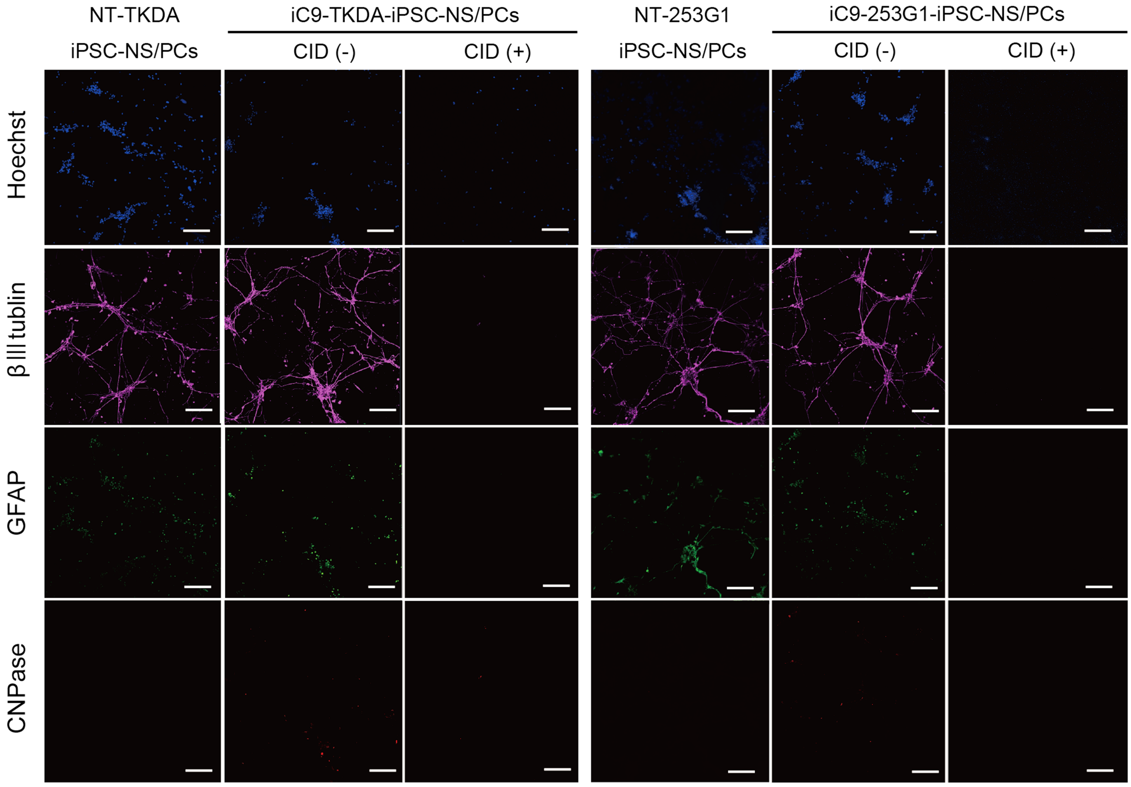

Figure S1. Integrated iC9 with CID-induced apoptosis in terminally differentiated iPSC derivatives

Terminally differentiated neurons and astrocytes from NT-iPSC-NS/PCs and iC9-iPSC-NS/PCs were treated with CID after 14 days of differentiation and stained. iC9-iPSC-NS/PCs differentiated into neurons and astrocytes similar to the NT-iC9-iPSC-NS/PCs. Activation of iC9 in these cells resulted in cellular apoptosis.

Scale bars = 100 μm.
