# Supplementary material for: Fail-Safe System against Potential Tumorigenicity after Transplantation of iPSC Derivatives
Source: Stem Cell Reports. 2017 Mar 2;8(3):673–84. doi: 10.1016/j.stemcr.2017.02.003 (PMC5355810; doi:10.1016/j.stemcr.2017.02.003)
Supplement: Document S2. Article plus Supplemental Information [file mmc2.pdf]

Stem Cell Reports
Article

OPEN ACCESS

Fail-Safe System against Potential Tumorigenicity after Transplantation of iPSC Derivatives

Go Itakura,[1,2] Soya Kawabata,[1,2] Miki Ando,[3] Yuichiro Nishiyama,[1,2] Keiko Sugai,[1,2] Masahiro Ozaki,[1,2] Tsuyoshi Iida,[1,2] Toshiki Ookubo,[1,2] Kota Kojima,[1,2] Rei Kashiwagi,[1,2] Kaori Yasutake,[1,2] Hiromitsu Nakauchi,[3] Hiroyuki Miyoshi,[1] Narihito Nagoshi,[2] Jun Kohyama,[1] Akio Iwanami,[2] Morio Matsumoto,[2] Masaya Nakamura,[2,*] and Hideyuki Okano[1,*]

[1]Department of Physiology, Keio University School of Medicine, 35 Shinanomachi, Shinjuku-ku, Tokyo 160-8582, Japan
[2]Department of Orthopaedic Surgery, Keio University School of Medicine, 35 Shinanomachi, Shinjuku-ku, Tokyo 160-8582, Japan
[3]Division of Stem Cell Therapy, Center for Stem Cell Biology and Regenerative Medicine, Institute of Medical Science, University of Tokyo, 4-6-1 Shirokanedai, Minato-ku, Tokyo 108-8639, Japan
*Correspondence: masa@a8.keio.jp (M.N.), hidokano@a2.keio.jp (H.O.)
http://dx.doi.org/10.1016/j.stemcr.2017.02.003

SUMMARY

Human induced pluripotent stem cells (iPSCs) are promising in regenerative medicine. However, the risks of teratoma formation and the overgrowth of the transplanted cells continue to be major hurdles that must be overcome. Here, we examined the efficacy of the inducible caspase-9 (iCaspase9) gene as a fail-safe against undesired tumorigenic transformation of iPSC-derived somatic cells. We used a lentiviral vector to transduce iCaspase9 into two iPSC lines and assessed its efficacy in vitro and in vivo. In vitro, the iCaspase9 system induced apoptosis in approximately 95% of both iPSCs and iPSC-derived neural stem/progenitor cells (iPSC-NS/PCs). To determine in vivo function, we transplanted iPSC-NS/PCs into the injured spinal cord of NOD/SCID mice. All transplanted cells whose mass effect was hindering motor function recovery were ablated upon transduction of iCaspase9. Our results suggest that the iCaspase9 system may serve as an important countermeasure against post-transplantation adverse events in stem cell transplant therapies.

INTRODUCTION

The establishment of induced pluripotent stem cells (iPSCs), which exhibit pluripotent differentiation and self-renewal potential comparable with that of embryonic stem cells (ESCs), by reprogramming via the introduction of several genes into somatic cells has opened up new prospects for regeneration medicine, and a number of possible clinical applications are currently being studied (Okano and Yamanaka, 2014; Takahashi et al., 2007). We previously reported the efficacy of the transplantation of human iPSC (hiPSC)-derived neural stem/progenitor cells (hiPSC-NS/PCs) in spinal cord-injured mice and common marmosets (Kobayashi et al., 2012; Nakamura and Okano, 2013; Nori et al., 2011). However, several groups have reported teratoma formation following the transplantation of ESC- or iPSC-derived products (Duinsbergen et al., 2009; Hentze et al., 2009; Seminatore et al., 2010). We have also observed the overgrowth of cellular grafts during long-term monitoring of transplanted hiPSC-NS/PCs (Nori et al., 2015). Thus, one of the greatest challenges in realizing clinical applications is the development of methodology to reduce the potential tumorigenicity of transplanted cells. Quality assessments of iPSCs and iPSC-derived products are now under way (Calderon et al., 2012; Denning et al., 2016; Doi et al., 2014; Hattori et al., 2010; Sundberg et al., 2013). Several attempts have been made to remove potentially tumorigenic cells during and after iPSC derivation. These have included attempts to remove residual undifferentiated iPSCs from the pre-transplanted cells by excluding glucose from the lactate-enriched medium (Tohyama et al., 2013), to extract target cells using surface antigen markers (Chen et al., 2011; Doi et al., 2014; Pruszak et al., 2009), to eliminate iPSCs by means of carbohydrate chains (Tateno et al., 2014, 2015), and to inhibit cellular overgrowth by treatment with γ-secretase prior to transplantation (Ogura et al., 2013; Okubo et al., 2016). Our group has also conducted studies involving the transplantation of cells that have been induced to differentiate into hiPSC-NS/PCs in immunodeficient animals to allow for the assessment of the cells prior to transplantation (Okano et al., 2013). Even if such attempts are successful, however, the risk of tumor formation in residual pluripotent stem cells below the threshold of detection cannot be completely eliminated. The precise mechanisms behind oncogenic transformation have not been clearly elucidated, although several mechanisms have been proposed, including differentiation of tumors into neural elements (Cai et al., 2010; Nori et al., 2015), tumor formation by cells with arrested differentiation, teratoma growth from remnant iPSCs, and previously unknown mechanisms of tumor formation, such as dedifferentiation (Kamada et al., 2016). It is impractical to identify all these risks through pre-clinical studies, and the development of pre-transplantation safety measures that eliminate all risk of tumorigenesis is unrealistic. For these reasons, post-transplantation safety measures, such as surgery and γ-ray

This is an open access article under the CC BY license (http://creativecommons.org/licenses/by/4.0/).

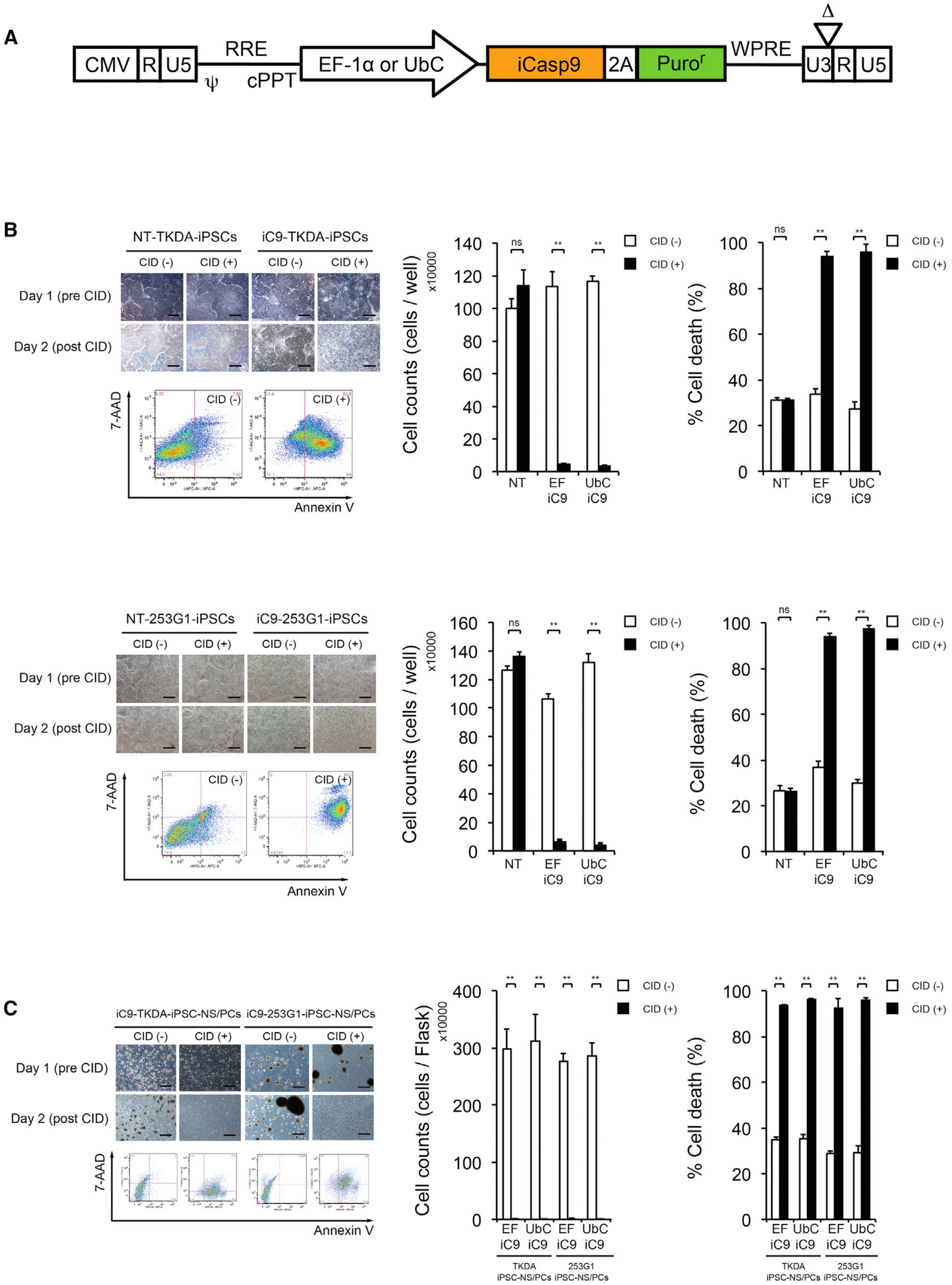

Figure 1. Integrated iC9 with CID Reliably Induced Apoptosis in the iPSCs, iPSC-NS/PCs, and Terminally Differentiated Derivatives
(A) Schematic representation of the lentiviral *iC9* bicistronic vector under the *EF-1α* or *Ubc* promoter. This vector contains the suicide gene *iC9*, a cleavable 2A-like sequence, and *Puro* as a selectable marker.

(legend continued on next page)

irradiation (Katsukawa et al., 2016), are also extremely important. In the present study, we explored the efficacy of a suicide gene introduced into iPSCs as a fail-safe against post-transplantation tumorigenic transformation.

A number of candidate suicide genes, such as HSV-TK, iCaspase9, human CD20, and mutant human thymidylate kinase, are currently being investigated for use in adoptive T cell therapy, which represents the front line of research regarding the establishment of gene-based fail-safe systems (Ciceri et al., 2009; Di Stasi et al., 2011; Griffioen et al., 2009; Introna et al., 2000; Marin et al., 2012). In studies of measures against tumorigenic transformation after ESC or iPSC transplantation, HSV-TK is the most commonly used gene (Chen et al., 2013; Cheng et al., 2012; Schuldiner et al., 2003; Zhao et al., 2012). However, apprehension has been expressed regarding the relatively poor ability of the HSV-TK inducer ganciclovir to cross the blood-brain barrier (BBB) (Nau et al., 2010), the immunogenicity of murine transgenes (Traversari et al., 2007), and the fact that it takes about 3 days to induce apoptosis using this approach (Ciceri et al., 2009). Moreover, there is a potential risk of adverse responses to long-term administration of ganciclovir, such as impairment of renal function, hepatic dysfunction, and pancytopenia, as well as secondary impairments caused by a bystander effect on adjacent tumors (Dachs et al., 2009; Zhao et al., 2012). Because ablation by the HSV-TK system is limited to cells with high proliferative potential (Brenner et al., 2013; Hoyos et al., 2012), its efficacy in slow-growing tumors and grafts that remain undifferentiated, which may trigger a mass effect due to confinement in the spinal cord, remains unclear. Fail-safe strategies involving the iCaspase9 gene are devoid of such immunogenic potential and induce apoptosis within 24 hr (Marin et al., 2012); such strategies have already been used to treat graft-versus-host disease following lymphocyte transplantation in clinical settings. The safety of this gene-based approach has been verified (Barese et al., 2015; Di Stasi et al., 2011), and it has received considerable attention for its potential in both gene transfer and the ablation of cells after transplantation (Ando et al., 2014, 2015). However, the clinical use of iCaspase9 in CNS disorders has been limited, and the trans-BBB intramedullary distribution after systemic injection of the apoptosis inducer, small-molecule chemical inducers of dimerization (CIDs), remains unclear. Its effect on post-iPSC transplantation teratomas has been demonstrated, but its effect on tumors arising from iPSC-derived somatic cells is unknown. In the present study, we evaluated the efficacy of the iCaspase9 system as a fail-safe system by introducing the system into hiPSCs and hiPSC-NS/PCs and transplanting the transduced cells into the injured spinal cords of mice.

RESULTS

Integrated iC9 with CID Reliably Induced Apoptosis in iPSCs, iPSC-NS/PCs, and Terminally Differentiated Derivatives

We prepared a lentiviral vector with the iCaspase9 gene (iC9) and the puromycin resistance gene as a selectable marker integrated under the EF-1α or UbC promoter (Figure 1A). The iCaspase9 gene was then transduced into two hiPSC lines with potential tumorigenicity after iPSC-NS/PCs transplantation in previous unpublished and published studies, TKDA3-4 and 253G1 (Nori et al., 2015). Cultures of these two iCaspase9-transduced hiPSC (iC9-iPSCs) lines and cultures of non-transduced iPSCs (NT-iPSCs) established by the addition of a small-molecule CID (AP20817) were incubated for 24 hr. After incubation, the cultures were subjected to cell morphological assessments, cell counting, and detection of apoptotic cells by flow cytometry after annexin V/7-AAD (7-aminoactinomycin D) viability staining (Figures 1B and 1C). In both cell lines, cellular detachment and a marked decrease in the viable cell counts were noted in the CID-added iC9-iPSC cultures (Figure 1B, left and middle). Fluorescence-activated cell sorting (FACS) analysis revealed that approximately 95% of the cells were apoptotic (Figure 1B, right). In the NT-iPSC or iC9-iPSC cultures grown in the absence of CID, on the other hand, the cells were found to be adherent to the dishes and maintained in colonies (Figure 1B, left and middle) with a cellular mortality rate as low as 30% (Figure 1B, right). We then investigated the effects of CID on hiPSC-NS/PCs derived by induced differentiation from these hiPSCs. Exposure to CID led to apoptosis of >93% of cells in the cultures of the iCaspase9-transduced hiPSC-NS/PCs (iC9-hiPSC-NS/PCs) in all cell lines tested (Figure 1C).

(B) Two iPSC lines, TKDA3-4 and 253G1, were transduced with lentiviral *EF-1α* or *UBC-iC9*. The transduced iC9-iPSC lines and non-transduced iPSC lines (NT-iPSC) were treated with CID, and apoptosis was measured 24 hr later by cell counting and flow cytometry for annexin V/7-AAD staining (n = 3 independent experiments). Values represent means ± SEM. **p < 0.0001 according to unpaired two-tailed Student's t tests and one-way ANOVA followed by the Tukey-Kramer test. ns, not significant. Scale bar, 1,000 μm.

(C) Two lines of iC9-iPSC-NS/PCs were treated with CID, and the degree of apoptosis was similar after 24 hr (n = 3 independent experiments). Values represent means ± SEM. **p < 0.0001 according to unpaired two-tailed Student's t tests and one-way ANOVA followed by the Tukey-Kramer test. ns, not significant. Scale bar, 1,000 μm.

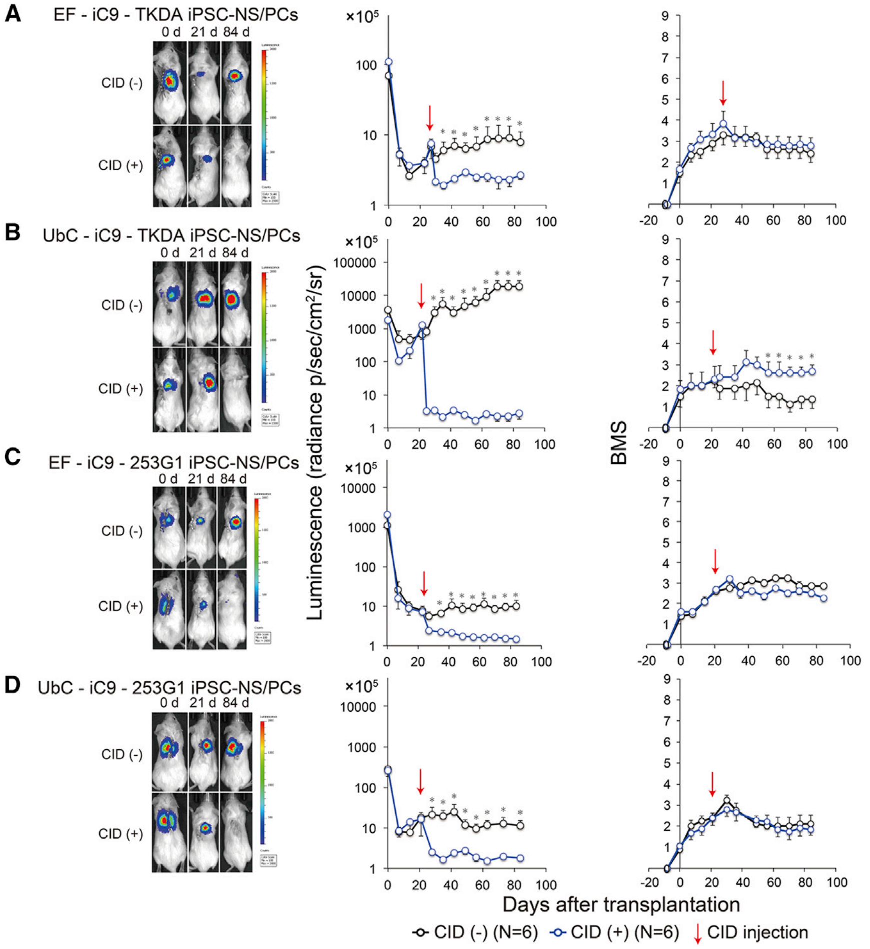

Figure 2. Integrated iC9 Ablated iPSC-NS/PC-Derived Tumors and Controlled Adverse Events after Transplantation

(A–D, Left) Bioluminescence images of representative mice at 0, 21, and 84 days after transplantation of iC9-iPSC-NS/PCs. Upper panel: an NOD/SCID mouse not treated with CID (CID(−) group); lower panel: an NOD/SCID mouse treated with CID (CID(+) group).

(A–D, Middle) Quantitative analysis of the photon counts derived from the grafted iC9-iPSC-NS/PCs. Values are expressed as means ± SEM. $^{*}p < 0.05$ according to one-way ANOVA followed by the Tukey-Kramer test.

(A–D, Right) Motor function in the hind limbs was assessed by BMS scores. Values are expressed as the means ± SEM. $^{*}p < 0.05$ according to one-way ANOVA followed by the Tukey-Kramer test.

N indicates the number of mice (i.e., n = 6 for each group).

Terminally differentiated neurons and astrocytes from hiPSC-NS/PCs were also similarly treated with CID after 14 days of induced differentiation, followed by immunostaining. There was no difference in the trends of differentiation between the NT-iPSC-NS/PCs and iC9-iPSC-NS/PCs following terminal induced differentiation, and treatment with CID also resulted in apoptosis of these terminally derived cells after induced differentiation (Figure S1). These results indicate that the iCaspase9 system exerts similar effects in hiPSCs, hiPSC-NS/PCs, neurons, and astrocytes following induced differentiation.

Integrated iC9 Abolished hiPSC-NS/PC-Derived Tumors and Controlled Adverse Events after Transplantation

We transplanted the two hiPSC-NS/PCs cell lines described above (TKDA3-4 and 253G1) into the injured spinal cords of NOD/SCID mice and followed their engraftment by performing weekly bioimaging checkups. The animals were dosed with CID when overt growth of the transplanted grafts was noted, and the treatment efficacy was assessed (Figure 2). Parallel observations included motor function assessments using the Basso Mouse Scale (BMS) scoring system and histological evaluations.

Transplantation of TKDA3-4 iPSC-NS/PCs

First, iC9-TKDA3-4 iPSC-NS/PCs were transplanted into the injured spinal cords of NOD/SCID mice. Luminescence began to increase from week 2 post transplantation in both the EF-iC9-TKDA3-4 iPSC-NS/PCs and UbC-iC9-TKDA3-4 iPSC-NS/PCs grafted groups. At week 3 post transplantation, the luminescence had increased to approximately 10% of that at the time of transplantation in the EF-iC9-TKDA3-4 hiPSC-NS/PCs grafted group, which was comparable with the level at the time of transplantation in the UbC-iC9-TKDA3-4 hiPSC-NS/PCs grafted group. The luminescence subsequently reached a plateau in the EF-iC9-TKDA3-4 hiPSC-NS/PCs grafted group, whereas in the UbC-iC9-TKDA3-4 hiPSC-NS/PCs grafted group it continued to increase until it reached 10-fold the initial level at week 12 post transplantation. In response to administration of CID at week 3 post transplantation, the

luminescence quickly diminished to the background level in all animals, even though the tumors exhibited rapid growth. Further observation revealed no evidence of a re-increase in the luminescence (Figures 2A and 2B, left and middle).

The hindlimb motor function of the mice increased slightly after transplantation in the EF-iC9-TKDA3-4 hiPSC-NS/PCs grafted group, but this improvement halted, followed by a gradual decrease. In the UbC-iC9-TKDA3-4 hiPSC-NS/PCs grafted group, the functional recovery was minimal immediately after transplantation, and a gradual functional decline was noted from week 2–3 post transplantation onward. Function eventually declined to below the baseline level. The motor function decreased following administration of CID in the EF-iC9-TKDA3-4 hiPSC-NS/PCs grafted group, although the subsequent decrease in BMS scores in this group was minimal compared with the scores in the non-CID-treated groups. In the UbC-iC9-TKDA3-4 hiPSC-NS/PCs grafted group, functional recovery was significantly increased after treatment with CID compared with that in the non-CID-treated groups at the final evaluation (Figures 2A and 2B, right).

Transplantation of 253G1 hiPSC-NS/PCs

We next transplanted iC9-253G1 hiPSC-NS/PCs into the injured spinal cords of NOD/SCID mice. Luminescence began to increase from week 2 post transplantation, reaching a plateau at week 3 post transplantation. In response to the administration of CID at this time, the luminescence rapidly diminished to the background level. Further observation revealed no evidence of a re-increase in the luminescence (Figures 2C and 2D, left and middle). Recovery of hindlimb motor function was noted, corresponding to an increase in the BMS score to 3 immediately following transplantation. The BMS score subsequently reached a plateau in the EF-iC9-253G1 hiPSC-NS/PCs grafted group. No depression of hindlimb motor function occurred thereafter in the present experiment. Although no significant difference was noted, motor function decreased slightly after administration of CID, and the BMS score tended to be lower in this group compared with the CID-treated groups at the final evaluation. The BMS score also increased to 3 or 4 after transplantation in the UbC-iC9-253G1 hiPSC-NS/PCs grafted group, although a subsequent gradual decline in the motor function was observed in this group. There was a slight depression of motor function after the administration of CID in the CID-treated groups. At the final observation, however, the BMS scores in the CID-treated groups showed no significant difference compared with those in the non-CID-treated groups, although they tended to be somewhat lower (Figures 2C and 2D, right).

Grafted hiPSC-NS/PCs Formed Neural Tumors and Teratomas, and the Activated iC9 System Induced Apoptosis in Both of These iPS-NS/PC-Derived Tumors

Enlarged grafts extending both cephalic and caudal directions in the spinal cord were noted in the EF-iC9-TKDA3-4 hiPSC-NS/PCs grafted group of mice (Figures 3A and 3B, left). Of the cells of the enlarged grafts, 19.2% were positive for Ki-67, up to 59.3% were positive for OCT4, and the majority of the cells were Nestin-positive undifferentiated neural precursor cells; the remaining small proportion of cells included cells that differentiated into the three neural linages (Figures 3C–3E, left). In the UbC-iC9-TKDA3-4 hiPSC-NS/PCs grafted group, on the other hand, all mice were found to bear giant tumors, which occasionally exhibited extramedullary spread, causing conspicuous displacement of the normal spinal cord parenchyma (Figures 3A and 3B, right). While the cells in some of these tumors were noted to have differentiated into neural linages, most tumors were found to be teratomas containing embryonic elements of all three primary germ cell layers. Immunohistological evaluations revealed that 23.3% of the cells were Ki-67 positive and 44.4% were OCT4 positive (Figures 3C–3E, right). At 1 week after the administration of CID all engrafted cells disappeared, similar to the results of the in vivo imaging system data. There was neither regrowth of the tumors nor grafted cell remnants, even after continued observation until week 12 post transplantation, similar to the outcome observed in the non-CID-treated groups (Figure 3).

In the EF-iC9-253G1 hiPSC-NS/PCs grafted group of mice, solid tumors composed of undifferentiated neural cells were found spreading through the entire spinal medulla, as previously reported (Figures 4A and 4B, left). Cellular elements of the graft were composed in parts of βIII-tubulin-positive neurons, glial fibrillary acidic protein (GFAP)-positive astrocytes, and adenomatous polyposis coli (APC)-positive oligodendrocytes, and the major part of the graft was composed of Nestin-positive cells. OCT4-positive cells accounted for 64.9% of cells and Ki-67-positive cells for 8.1% of cells (Figures 4C–4E, left). One week after the administration of CID, a major portion of the graft disappeared. There were no cellular remnants at the final observation.

The graft presented features of a tumor largely composed of Nestin-positive undifferentiated neural cells in the UbC-iC9-253G1 hiPSC-NS/PCs grafted group, as in the EF-iC9-253G1 hiPSC-NS/PCs grafted group. Occasionally, extramedullary tumor formation was observed (Figures 4A and 4B, right). OCT4-positive cells made up 54.0% of all cells, and Ki-67-positive cells accounted for 11.0% of all cells (Figures 4C–4E, right). Following CID treatment, all of the transplanted cells disappeared.

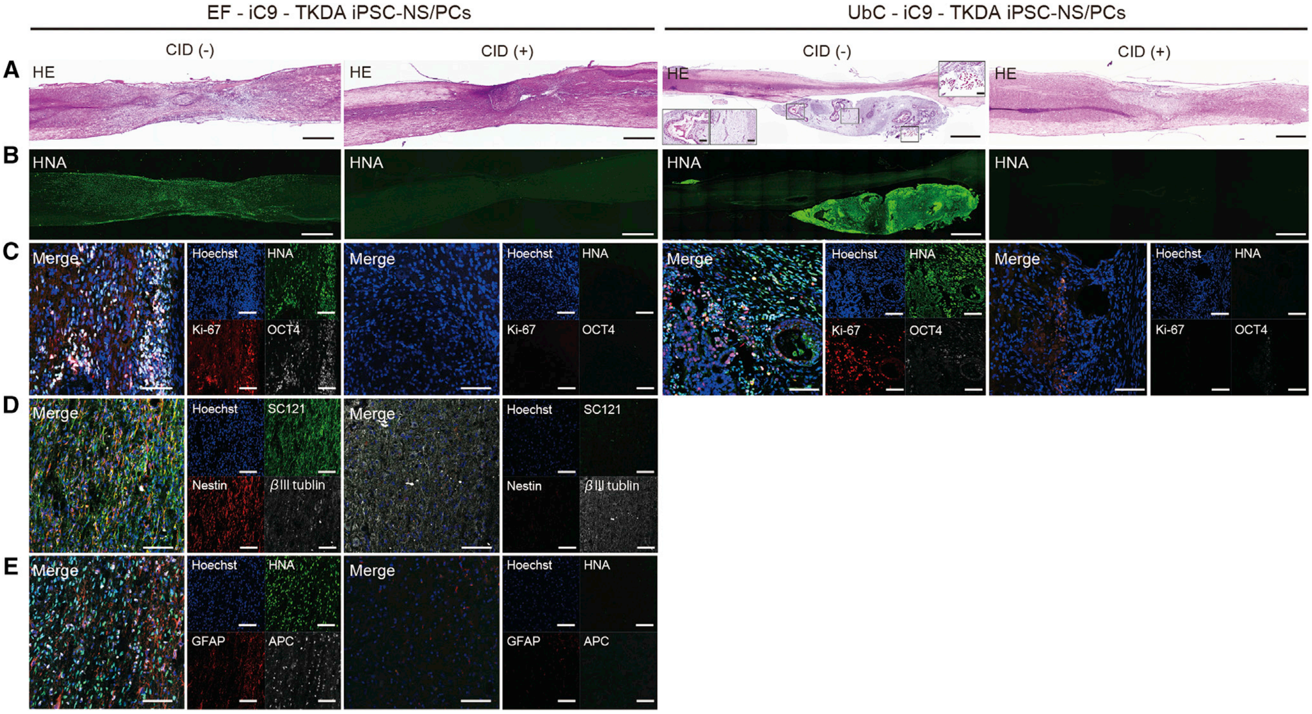

Figure 3. Grafted TKDA3-4 iPSC-NS/PCs Formed Neural Tumors and Teratomas, and the Activated iC9 System Induced Apoptosis of Both of these iPSC-NS/PC-Derived Tumors

(A and B) Representative sagittal sections of the spinal cord stained with H&E (A) and human nuclear antigen (HNA) (B) at 84 days after cell transplantation.

(C–E) Representative images of the grafted cells immunostained for Hoechst/HNA/Ki-67/OCT (C), Hoechst/Stem 121/Nestin/βIII tubulin (D), and Hoechst/HNA/glial fibrillary acidic protein (GFAP)/APC (E).

Scale bars, 500 μm (A and B) and 100 μm (C–E).

DISCUSSION

In the present study, we examined the apoptosis-inducing efficacy of iC9-TKDA3-4-hiPSC-NS/PCs and iC9-253G1 hiPSC-NS/PCs in vitro. Both showed a high propensity for oncogenic transformation after transplantation. The cellular grafts were then transplanted into injured spinal cords of NOD/SCID mice to allow for tumor formation. Our results demonstrate the efficacy of the iCaspase9 system in inducing apoptosis in both intraspinal cord teratomas and intraspinal cord neural tumors.

We successfully achieved quick induction of apoptosis in approximately 95% of the iPSCs in vitro, as in a previous report (Ando et al., 2015). This apoptosis induction rate was essentially comparable with the data reported from experiments carried out using the HSV-TK system (Chen et al., 2013). We observed iCaspase9 gene expression in all cell types that were examined, including the hiPSC-NS/PCs and their terminally differentiated products, namely, neurons. Furthermore, we found that there was no detectable influence of the iCaspase9 gene system on the efficiency of terminal differentiation of hiPSC-NS/PCs into neurons and astrocytes. No significant differences were observed between the EF-iC9-iPSCs and UbC-iC9-iPSCs or between the EF-iC9-iPS-NS/PCs and UbC-iC9-iPS-NS/PCs, although the rate of apoptosis induction was somewhat higher in the UbC-iC9-iPS-NS/PCs. Reports of studies of iCaspase9 gene transduction under the EF1α promoter revealed that DNA methylation occurs during the process of differentiation, which results in downregulation of the iCaspase9 gene expression (Wu et al., 2014) or iCaspase9 gene silencing (Pfaff et al., 2013). Contaminant non-iCaspase9-expressing cells may have had a lower rate of apoptosis induction in the EF-iC9 iPSC and hiPSC-NS/PC populations in the present study via the same mechanism. We performed further observation by serial subculturing after exposure to CID, whereby no viable cells were observed. However, this may not represent complete killing, because annexin V/7-AAD-positive cells failed to reach 100%. Therefore, new genetic manipulation technology that ensures gene insertion and enhances the effectiveness of the iCaspase9 system is required to be widely used.

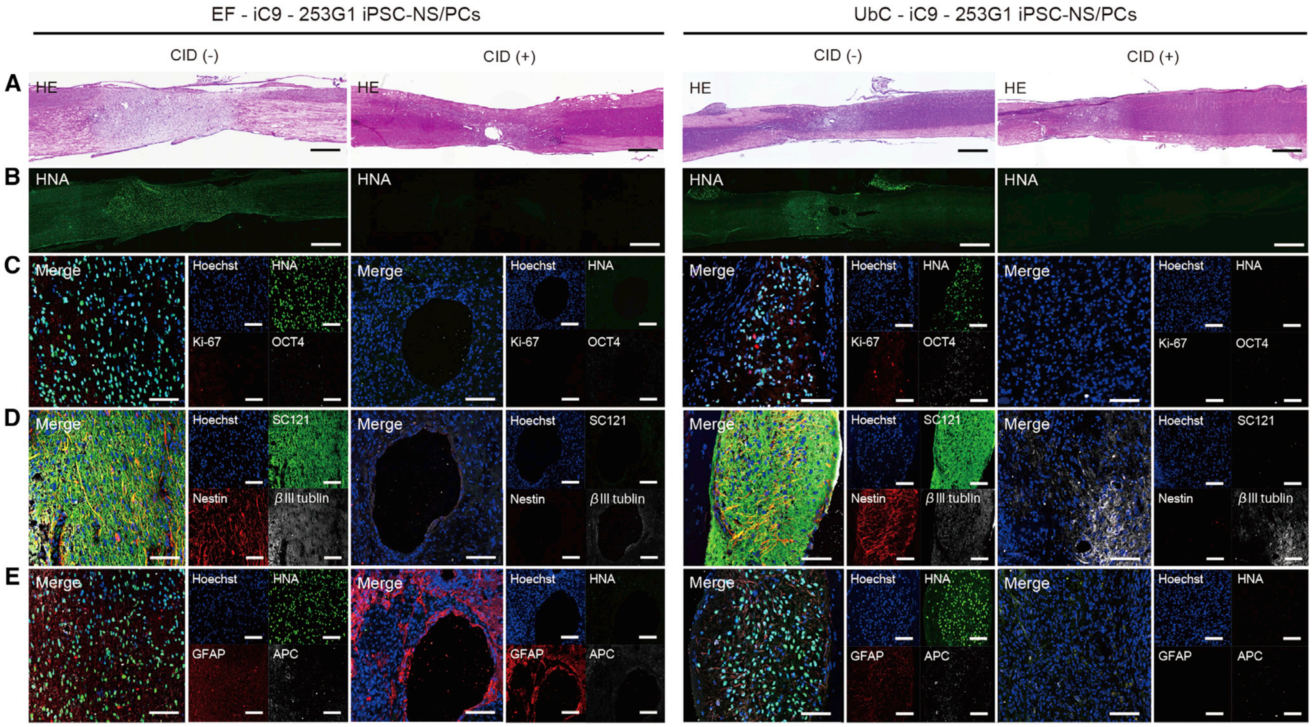

Figure 4. Engrafted 253G1 iPSC-NS/PCs Formed Neural Tumors, and the Activated iC9 System Induced Apoptosis in the 253G1 iPSC-NS/PC-Derived Tumors

(A and B) Representative sagittal sections of a spinal cord stained with H&E (A) and human nuclear antigen (HNA) (B) at 84 days after cell transplantation.

(C–E) Representative images of the grafted cells immunostained for Hoechst/HNA/Ki-67/OCT (C), Hoechst/Stem 121/Nestin/βIII tubulin (D), and Hoechst/HNA/glial fibrillary acidic protein (GFAP)/APC (E).

Scale bars, 500 μm (A and B) and 100 μm (C–E).

We also evaluated the apoptosis-inducing effect of iCaspase9 in vivo. Tumorous proliferation of diverse histological types was observed following grafting of the hiPSC-NS/PCs. In all of the EF-iC9-TKDA3-4 iPS-NS/PCs, EF-iC9-253G1 hiPSC-NS/PCs, and UbC-iC9-253G1 hiPSC-NS/PCs grafted groups in this study, non-teratomatous neural tumor formation occurred, consistent with previous reports (Itakura et al., 2015; Nori et al., 2015). Although a larger portion of the tumors exhibited solid growth of Nestin-positive cells that had not terminally differentiated, some of the transplanted cells had differentiated into neurons, astrocytes, or oligodendrocytes. In the UbC-iC9-TKDA3-4 hiPSC-NS/PCs grafted group, however, the graft rapidly increased in size, growing into a teratoma containing embryonic elements of all three primary germ cell layers. In the apoptosis-induced groups, administration of CID was followed by quick disappearance of all the transplanted cells, regardless of the type of tumor formed, and there was neither gross evidence of graft re-enlargement nor histological evidence of remnant cells, even at long-term follow-up. It had previously been unclear whether systemically administered CID is distributed into the cerebrospinal fluid; however, in the present study we found that intraperitoneally injected CID rapidly reached cells engrafted in the CNS and induced apoptosis, similar to reports of previous studies of subcutaneous tissue and blood. This suggests that CID is an effective inducer even in the CNS protected by the BBB. In the present study, nevertheless, the CID treatment was undertaken after tumor formation. Thus, it is likely that pronounced neovascularization was present in the peritumoral region at the point of CID treatment, which would result in efficient induction of apoptosis in the presence of the undeveloped newly formed BBB. Further discussion of CID permeability through the BBB is needed in the future.

The EF-iC9-TKDA3-4 hiPSC-NS/PCs, EF-iC9-253G1 hiPSC-NS/PCs, and UbC-iC9-253G1 hiPSC-NS/PCs grafted mice exhibited improved hindlimb motor function until week 4 after transplantation, with a subsequent gradual decrease of function. In the CID-treated groups there was a slight decline in function followed by a plateau. In the UbC-iC9-TKDA3-4 iPS-NS/PC-grafted mice, compared

with hindlimb function at the time of the transplantation, hindlimb function declined due to enlargement of the teratoma to the extent that the ankle joint was slightly movable but barely capable of bearing weight. In the CID-treated groups, on the other hand, no such deterioration due to tumor enlargement was noted after the disappearance of the transplanted cell-derived tumors in response to CID injection, and there was a slight recovery of function compared with function at the time of transplantation. However, this was comparable with the hindlimb motor function observed in the PBS-grafted groups reported in previous studies and may represent spontaneous progression post spinal cord injury in NOD/SCID mice (Nori et al., 2011). According to previous studies in which tumorigenic transformation was not observed post transplantation, transplanted cells appeared to contribute to the improvement of motor function (Fujimoto et al., 2012; Nori et al., 2011). When these cells that contributed to the functional recovery were ablated, motor function declined, but there was a slight recovery in the final motor function compared with the PBS-transplanted groups (Abematsu et al., 2010). Owing to the intact motor function in the mice of the transplanted cell ablation group among the present iC9-TKDA3-4 hiPSC-NS/PCs grafted groups, it did not appear that the iC9-TKDA3-4 hiPSC-NS/PCs were able to improve hindlimb motor function through mechanisms such as reconstruction of neural circuits in the injured spinal cord after the transplantation. Furthermore, it has been reported that hiPSC-NS/PCs liberate a variety of humoral factors (Kobayashi et al., 2012). Our data showed no evidence of functional recovery attributable to humoral factors after transplantation of the UbC-iC9-TKDA3-4 hiPSC-NS/PCs that formed teratomas. Eventually, a gradual depression of hindlimb function due to a mass effect associated with tumor enlargement was observed. Subsequently, we noted that ablation of the transplanted cells prevented the depression of hindlimb function, leading to a restoration to spontaneous progression after spinal cord injury. For the iC9-253G1 hiPSC-NS/PCs grafted groups, on the other hand, a slight decrease in hindlimb function was observed in the CID-treated group, which suggests that the treatment may contribute to functional recovery even in the presence of abnormal proliferation of undifferentiated neural cells. Furthermore, changes in motor function associated with the rapidly growing tumors, such as teratomas, were reversible provided that the duration of tissue displacement by the tumor was <3 weeks, and full rescue from tumor-related adverse events by administration of CID seems achievable. Previous assessments have shown that, in the case of slowly growing tumors in the spinal cord, hindlimb motor function begins to decline due to tumor enlargement and progresses to complete paralysis. However, in our study, recovery from depressed hindlimb motor function was evident even after tumor ablation on day 100 post transplantation (Itakura et al., 2015). We cannot exclude the possibility, however, that additional functional depression caused by a rapidly growing tumor may appear during longer-term follow-up.

The aim of the present study was to explore the induction of apoptosis in tumors formed from hiPSC-derived products. Our results demonstrate that apoptosis can be induced by CID in tumors grown from transplanted cells that have undergone differentiation after engraftment and have been in close interaction with host tissues independent of the underlying mechanism of tumor formation and differentiation. Our results also showed that injected CID is able to reach transplanted cells and induce their apoptosis even in the spinal cord, which is segregated from the bloodstream by the BBB, just as in subcutaneous tissues and circulating blood. We also confirmed that the administered CID inhibited the growth of tumors formed after cell transplantation, and quickly and completely ablated the transplanted cells. In the future, it may become feasible to specifically conserve differentiated neural cells by integrating a suicide gene under a tumorigenesis-specific promoter using a factor such as the HSV-TK gene rather than by means of total ablation. This will allow for a more selective ablation of tumor-initiating cells and proliferative cells. Under the present conditions, whereby the conceivable mechanisms of tumorigenic transformation are diverse and other risks involved in the transplantation of cells remain unclear, methods for completely ablating or "undoing" all possible adverse events must be an option. In the present study, the iCaspase9 system was shown to be able to induce apoptosis in a broad range of cell and tissue types, including hiPSCs, differentiated hiPSC-NS/PCs, neurons, teratomas formed from hiPSC-derived products, and differentiated neural tumors. We also found no evidence of remnant cells on long-term follow-up, suggesting the ablation, rather than the involution, of tumors by the iCaspase9 system. Furthermore, iCaspase9 gene transfection into good clones may lead to elucidation of the neural regeneration mechanism of transplanted cells. We recognize that the transduction of the iCaspase9 system using a lentiviral vector represents a potential shortcoming of the present study. There are still a number of problems that need to be resolved, and at present it is still not suitable for clinical application. The use of a non-integrating vector (Uno et al., 2015) or gene introduction into a particular gene locus, e.g., a safety harbor, would help to minimize the risks associated with gene introduction by transfection. Furthermore, the potential effects of iCaspase9 gene on the differentiation and characteristics of the transplanted cells cannot be ruled out; hence, long-term follow-up is necessary. Once these issues are resolved, the iCaspase9 system

may become a useful method for reducing risk in clinical applications of hiPSC-derived products.

EXPERIMENTAL PROCEDURES

hiPSC Culture and Lentiviral iC9 Transduction

Cell cultures of hiPSCs (hiPSC clone 253G1 [Nakagawa et al., 2008] and TKDA3-4 [Yanagida et al., 2013]) were established as previously described (Itakura et al., 2015) with slight modifications. For introduction of the iC9 gene into the iPSCs, 1 × 10^5 iPSCs were plated on 6-well plates coated with iMatrix-511 (Nippi) and cultured in StemFit AK03 medium (Ajinomoto) for 24 hr. The iPSCs were then transduced with the prepared iCaspase9-expressing lentiviral vectors at an MOI of 20 according to a previously reported method (Ando et al., 2015). The medium was replaced every day after transduction. iCaspase9-expressing cells were selected with puromycin-containing medium (1 μg/mL, Sigma P9620).

Neural Induction and Lentiviral ffLuc Transduction

Neural induction was performed as described in a previous report (Itakura et al., 2015) with slight modifications. For generation of hiPSC-NS/PCs, embryoid bodies (EBs) were generated from iPSCs grown in suspension in bacterial culture dishes without fibroblast growth factor 2 (FGF-2) for 4 weeks. The EBs were next dissociated into single cells using TrypLE Select and cultured in suspension at 1 × 10^6 cells/mL in media hormone mix supplemented with B27 and 20 ng/mL of FGF-2 (PeproTech) and 10 ng/mL hLif (Merck) with 1 μg/mL puromycin for 12 days. The ffLuc lentivirus was prepared and transduced into neurospheres following a method described in a previous report (Itakura et al., 2015). In brief, primary neurospheres were dissociated and transduced with lentivirus-expressing ffLuc (Venus fused to firefly luciferase) (Hara-Miyauchi et al., 2012) under the control of an elongation factor promoter (pCS II-EF-dVenus-Luc2). These primary neurospheres were passaged to fourth-passage neurospheres and used for transplantation.

Measurement of Apoptosis

Eighty nmol/L CID/AP20187 (Clontech) was added to a dish containing NT-iC9-hiPSCs or a flask containing iC9-hiPSC-NS/PCs. Twenty-four hours after CID addition, the cells were stained with annexin V (BD Biosciences) and 7-AAD (BD Biosciences) for 15 min according to the manufacturer's instructions. Flow cytometry was performed using a FACS Verse instrument (BD Biosciences) with FlowJo software (Tree Star).

Spinal Cord Injury Model and Transplantation of iPS-NS/PCs

Eight-week-old female NOD-SCID mice (20–22 g) were anesthetized by an intraperitoneal injection of ketamine (100 mg/kg) and xylazine (10 mg/kg). After laminectomy at the tenth thoracic spinal vertebra, the dorsal surface of the dura mater was exposed. Contusive spinal cord injury was induced at the Th10 level using an IH impactor (60 kdyn; Precision Systems and Instrumentation). Nine days after the injury was induced, 5 × 10^5 iC9-iPSC-NS/PCs were transplanted into the lesion epicenter of each mouse (n = 48) using a glass micropipette at a rate of 1 μL/min using a 25-μL Hamilton syringe and a stereotaxic microinjector (KDS 310, Muromachi Kikai). All experiments were performed in accordance with the Guidelines for the Care and Use of Laboratory Animals of Keio University (Assurance No. 13020) and the Guide for the Care and Use of Laboratory Animals (NIH). All surgeries were performed under anesthesia, efforts were made to minimize animal suffering, and humane endpoints were used.

Motor Function Analyses

Hindlimb motor function was evaluated after the induction of contusive spinal cord injury according to the BMS locomotor rating scale. Well-trained investigators blinded to the treatments performed the behavioral analyses.

Bioluminescence Imaging

The Xenogen-IVIS spectrum cooled charge-coupled device optical macroscopic imaging system (Caliper Life-Sciences) was used for the bioluminescence imaging (BLI), which was performed to confirm the survival of the transplanted iPSC-NS/PCs. Monitoring was performed once per week after cell transplantation. In brief, D-luciferin (Promega) was administered via intraperitoneal injection at a dose of 300 mg/kg body weight. Animals were placed in a light-tight chamber, and photons emitted from the luciferase-expressing cells were collected with integration times of 5 s to 2 min depending on the intensity of the bioluminescence emission. BLI signals were quantified in maximum radiance units (photons per second per centimeter squared per steradian [p/s/cm^2/sr]) and presented as log_{10}(photons per second).

Induced Apoptosis of iPS-NS/PC-Derived Tumor

Tumor-loaded mice were treated with CID (50 μg via intraperitoneal injection daily for 3 days) (n = 24). The CID(−) group mice that were not administered CID were used as control mice (n = 24).

Histological Analysis

Animals were anesthetized and transcardially perfused with 0.1 M PBS containing 4% paraformaldehyde. The spinal cords were removed, embedded in Optimal Cutting Temperature compound (Sakura FineTechnical), and sectioned in the sagittal plane using a cryostat (Leica CM3050 S, Leica Microsystems). Sections were stained with H&E, Hoechst 33258 dye (10 μg/mL; Sigma-Aldrich B2883), and the following primary antibodies: anti-GFP (rabbit immunoglobulin G [IgG], 1:200; Frontier Institute Af2020), anti-β-tubulin isotype III (mouse IgG, 1:1,000; Sigma-Aldrich T8660), anti-GFAP (rabbit IgG, 1:200; Dako Z0334), anti-CNPase (mouse IgG, 1:1,000; Sigma-Aldrich C5922), anti-HNA (mouse IgG, 1:300; Chemicon MAB1281), anti-human cytoplasm (STEM121) (mouse IgG, 1:200; StemCells Y40410), anti-human-specific Nestin protein (rabbit IgG, 1:200; described previously [Iwanami et al., 2005; Kanemura et al., 2002]), anti-Ki-67 (rabbit IgG, 1:200; Leica Biosystems PA0230), anti-OCT4 (mouse IgG, 1:50; Santa Cruz Biotechnology SC5279), and anti-APC CC-1 (mouse IgG, 1:200; Abcam ab16794). Samples were examined under an inverted fluorescence microscope (BZ 9000, Keyence) or confocal laser scanning microscope (LSM 700, Carl Zeiss). For quantification of the human nuclear antigen (HNA)-, Ki-67-, and OCT4-positive cells, three

representative mid-sagittal sections were selected, and five regions within 1 mm rostral and caudal to the lesion epicenter were automatically captured at 200× magnification (n = 3 per group). The numbers of marker-positive cells were counted in each section.

Statistical Analysis

All data are presented as mean ± SEM. Apoptosis was analyzed by performing an unpaired two-tailed Student's t test and one-way ANOVA followed by the Tukey-Kramer test. Differences in the quantitative analysis of the photon counts and BMS were analyzed by one-way ANOVA followed by the Tukey-Kramer test. All statistical analyses were performed using the Prism (GraphPad Software) programs. For all statistical analyses shown in the figures, the significance level was set at $^*p < 0.05$ and $^{**}p < 0.0001$.

SUPPLEMENTAL INFORMATION

Supplemental Information includes one figure and can be found with this article online at http://dx.doi.org/10.1016/j.stemcr.2017.02.003.

AUTHOR CONTRIBUTIONS

G.I.: conception and design, experiments, data analysis and interpretation, manuscript preparation, and final approval of manuscript; S.K., M.A., Y.N., K.S., M.O., T.I., T.O., K.K., R.K., K.Y., and H.M.: experiments, surgery, and data analysis and interpretation; H.N., N.N., J.K., A.I., and M.M.: conception and design, and data analysis and interpretation; M.N. and H.O.: conception and design, data analysis and interpretation, manuscript preparation, and final approval of manuscript.

ACKNOWLEDGMENTS

We appreciate the help of Dr. S. Shibata, Dr. F. Renault-Mihara, Dr. M. Shinozaki, Dr. S. Tashiro, Dr. K. Matsubayashi, Dr. K. Ito, Dr. Y. Tanimoto, Dr. Y. Hoshino, Dr. Y. Fukushima, and Ms. M. Isoda who are all members of the spinal cord research team at the Department of Physiology, Orthopedic Surgery and Rehabilitation Medicine, Keio University School of Medicine, Tokyo, Japan. We also thank Prof. Douglass Sipp (Keio University) for invaluable comments on the manuscript and Ms. T. Harada for assistance with animal care. We also thank Dr. S. Yamanaka and M. Ando for the human iPSC clones (253G1 and TKDA3-4). This work was supported by the Research Center Network for Realization of Regenerative Medicine by the Japan Science and Technology Agency (JST) and the Japan Agency for Medical Research and Development (AMED) (grant no. 16bm0204001h0004 to H.O. and M.N.), a Grant-in-Aid for Scientific Research by Japan Society for the Promotion of Science (grant no. 15K10422 to A.I.), and a grant by The General Insurance Association of Japan (grant no. 15-2B-13 to A.I.). H.O. is a founding scientist and a paid SAB of San Bio, Co., Ltd.

Received: October 31, 2016
Revised: February 2, 2017
Accepted: February 2, 2017
Published: March 2, 2017

REFERENCES

Abematsu, M., Tsujimura, K., Yamano, M., Saito, M., Kohno, K., Kohyama, J., Namihira, M., Komiya, S., and Nakashima, K. (2010). Neurons derived from transplanted neural stem cells restore disrupted neuronal circuitry in a mouse model of spinal cord injury. J. Clin. Invest. *120*, 3255–3266.

Ando, M., Hoyos, V., Yagyu, S., Tao, W., Ramos, C.A., Dotti, G., Brenner, M.K., and Bouchier-Hayes, L. (2014). Bortezomib sensitizes non-small cell lung cancer to mesenchymal stromal cell-delivered inducible caspase-9-mediated cytotoxicity. Cancer Gene Ther. *21*, 472–482.

Ando, M., Nishimura, T., Yamazaki, S., Yamaguchi, T., Kawana-Tachikawa, A., Hayama, T., Nakauchi, Y., Ando, J., Ota, Y., Takahashi, S., et al. (2015). A safeguard system for induced pluripotent stem cell-derived rejuvenated T cell therapy. Stem Cell Rep. *5*, 597–608.

Barese, C.N., Felizardo, T.C., Sellers, S.E., Keyvanfar, K., Di Stasi, A., Metzger, M.E., Krouse, A.E., Donahue, R.E., Spencer, D.M., and Dunbar, C.E. (2015). Regulated apoptosis of genetically modified hematopoietic stem and progenitor cells via an inducible caspase-9 suicide gene in rhesus macaques. Stem Cells *33*, 91–100.

Brenner, M.K., Gottschalk, S., Leen, A.M., and Vera, J.F. (2013). Is cancer gene therapy an empty suit? Lancet Oncol. *14*, e447–456.

Cai, J., Yang, M., Poremsky, E., Kidd, S., Schneider, J.S., and Iacovitti, L. (2010). Dopaminergic neurons derived from human induced pluripotent stem cells survive and integrate into 6-OHDA-lesioned rats. Stem Cells Dev. *19*, 1017–1023.

Calderon, D., Planat-Benard, V., Bellamy, V., Vanneaux, V., Kuhn, C., Peyrard, S., Larghero, J., Desnos, M., Casteilla, L., Puceat, M., et al. (2012). Immune response to human embryonic stem cell-derived cardiac progenitors and adipose-derived stromal cells. J. Cell Mol. Med. *16*, 1544–1552.

Chen, H.F., Chuang, C.Y., Lee, W.C., Huang, H.P., Wu, H.C., Ho, H.N., Chen, Y.J., and Kuo, H.C. (2011). Surface marker epithelial cell adhesion molecule and E-cadherin facilitate the identification and selection of induced pluripotent stem cells. Stem Cell Rev. *7*, 722–735.

Chen, F., Cai, B., Gao, Y., Yuan, X., Cheng, F., Wang, T., Jiang, M., Zhou, Y., Lahn, B.T., Li, W., et al. (2013). Suicide gene-mediated ablation of tumor-initiating mouse pluripotent stem cells. Biomaterials *34*, 1701–1711.

Cheng, F., Ke, Q., Chen, F., Cai, B., Gao, Y., Ye, C., Wang, D., Zhang, L., Lahn, B.T., Li, W., et al. (2012). Protecting against wayward human induced pluripotent stem cells with a suicide gene. Biomaterials *33*, 3195–3204.

Ciceri, F., Bonini, C., Stanghellini, M.T., Bondanza, A., Traversari, C., Salomoni, M., Turchetto, L., Colombi, S., Bernardi, M., Peccatori, J., et al. (2009). Infusion of suicide-gene-engineered donor lymphocytes after family haploidentical haemopoietic stem-cell transplantation for leukaemia (the TK007 trial): a non-randomised phase I-II study. Lancet Oncol. *10*, 489–500.

Dachs, G.U., Hunt, M.A., Syddall, S., Singleton, D.C., and Patterson, A.V. (2009). Bystander or no bystander for gene directed enzyme prodrug therapy. Molecules *14*, 4517–4545.

Denning, C., Borgdorff, V., Crutchley, J., Firth, K.S., George, V., Kalra, S., Kondrashov, A., Hoang, M.D., Mosqueira, D., Patel, A.,

et al. (2016). Cardiomyocytes from human pluripotent stem cells: from laboratory curiosity to industrial biomedical platform. Biochim. Biophys. Acta *1863*, 1728–1748.

Di Stasi, A., Tey, S.K., Dotti, G., Fujita, Y., Kennedy-Nasser, A., Martinez, C., Straathof, K., Liu, E., Durett, A.G., Grilley, B., et al. (2011). Inducible apoptosis as a safety switch for adoptive cell therapy. N. Engl. J. Med. *365*, 1673–1683.

Doi, D., Samata, B., Katsukawa, M., Kikuchi, T., Morizane, A., Ono, Y., Sekiguchi, K., Nakagawa, M., Parmar, M., and Takahashi, J. (2014). Isolation of human induced pluripotent stem cell-derived dopaminergic progenitors by cell sorting for successful transplantation. Stem Cell Rep. *2*, 337–350.

Duinsbergen, D., Salvatori, D., Eriksson, M., and Mikkers, H. (2009). Tumors originating from induced pluripotent stem cells and methods for their prevention. Ann. N. Y. Acad. Sci. *1176*, 197–204.

Fujimoto, Y., Abematsu, M., Falk, A., Tsujimura, K., Sanosaka, T., Juliandi, B., Semi, K., Namihira, M., Komiya, S., Smith, A., et al. (2012). Treatment of a mouse model of spinal cord injury by transplantation of human induced pluripotent stem cell-derived long-term self-renewing neuroepithelial-like stem cells. Stem Cells *30*, 1163–1173.

Griffioen, M., van Egmond, E.H., Kester, M.G., Willemze, R., Falkenburg, J.H., and Heemskerk, M.H. (2009). Retroviral transfer of human CD20 as a suicide gene for adoptive T-cell therapy. Haematologica *94*, 1316–1320.

Hara-Miyauchi, C., Tsuji, O., Hanyu, A., Okada, S., Yasuda, A., Fukano, T., Akazawa, C., Nakamura, M., Imamura, T., Matsuzaki, Y., et al. (2012). Bioluminescent system for dynamic imaging of cell and animal behavior. Biochem. Biophys. Res. Commun. *419*, 188–193.

Hattori, F., Chen, H., Yamashita, H., Tohyama, S., Satoh, Y.S., Yuasa, S., Li, W., Yamakawa, H., Tanaka, T., Onitsuka, T., et al. (2010). Nongenetic method for purifying stem cell-derived cardiomyocytes. Nat. Methods *7*, 61–66.

Hentze, H., Soong, P.L., Wang, S.T., Phillips, B.W., Putti, T.C., and Dunn, N.R. (2009). Teratoma formation by human embryonic stem cells: evaluation of essential parameters for future safety studies. Stem Cell Res. *2*, 198–210.

Hoyos, V., Savoldo, B., and Dotti, G. (2012). Genetic modification of human T lymphocytes for the treatment of hematologic malignancies. Haematologica *97*, 1622–1631.

Introna, M., Barbui, A.M., Bambacioni, F., Casati, C., Gaipa, G., Borleri, G., Bernasconi, S., Barbui, T., Golay, J., Biondi, A., et al. (2000). Genetic modification of human T cells with CD20: a strategy to purify and lyse transduced cells with anti-CD20 antibodies. Hum. Gene Ther. *11*, 611–620.

Itakura, G., Kobayashi, Y., Nishimura, S., Iwai, H., Takano, M., Iwanami, A., Toyama, Y., Okano, H., and Nakamura, M. (2015). Controlling immune rejection is a fail-safe system against potential tumorigenicity after human iPSC-derived neural stem cell transplantation. PLoS One *10*, e0116413.

Iwanami, A., Kaneko, S., Nakamura, M., Kanemura, Y., Mori, H., Kobayashi, S., Yamasaki, M., Momoshima, S., Ishii, H., Ando, K., et al. (2005). Transplantation of human neural stem cells for spinal cord injury in primates. J. Neurosci. Res. *80*, 182–190.

Kamada, M., Mitsui, Y., Matsuo, T., and Takahashi, T. (2016). Reversible transformation and de-differentiation of human cells derived from induced pluripotent stem cell teratomas. Hum. Cell *29*, 1–9.

Kanemura, Y., Mori, H., Kobayashi, S., Islam, O., Kodama, E., Yamamoto, A., Nakanishi, Y., Arita, N., Yamasaki, M., Okano, H., et al. (2002). Evaluation of in vitro proliferative activity of human fetal neural stem/progenitor cells using indirect measurements of viable cells based on cellular metabolic activity. J. Neurosci. Res. *69*, 869–879.

Katsukawa, M., Nakajima, Y., Fukumoto, A., Doi, D., and Takahashi, J. (2016). Fail-safe therapy by gamma-ray irradiation against tumor formation by human-induced pluripotent stem cell-derived neural progenitors. Stem Cells Dev. *25*, 815–825.

Kobayashi, Y., Okada, Y., Itakura, G., Iwai, H., Nishimura, S., Yasuda, A., Nori, S., Hikishima, K., Konomi, T., Fujiyoshi, K., et al. (2012). Pre-evaluated safe human iPSC-derived neural stem cells promote functional recovery after spinal cord injury in common marmoset without tumorigenicity. PLoS One *7*, e52787.

Marin, V., Cribioli, E., Philip, B., Tettamanti, S., Pizzitola, I., Biondi, A., Biagi, E., and Pule, M. (2012). Comparison of different suicide-gene strategies for the safety improvement of genetically manipulated T cells. Hum. Gene Ther. Methods *23*, 376–386.

Nakagawa, M., Koyanagi, M., Tanabe, K., Takahashi, K., Ichisaka, T., Aoi, T., Okita, K., Mochiduki, Y., Takizawa, N., and Yamanaka, S. (2008). Generation of induced pluripotent stem cells without Myc from mouse and human fibroblasts. Nat. Biotechnol. *26*, 101–106.

Nakamura, M., and Okano, H. (2013). Cell transplantation therapies for spinal cord injury focusing on induced pluripotent stem cells. Cell Res. *23*, 70–80.

Nau, R., Sorgel, F., and Eiffert, H. (2010). Penetration of drugs through the blood-cerebrospinal fluid/blood-brain barrier for treatment of central nervous system infections. Clin. Microbiol. Rev. *23*, 858–883.

Nori, S., Okada, Y., Yasuda, A., Tsuji, O., Takahashi, Y., Kobayashi, Y., Fujiyoshi, K., Koike, M., Uchiyama, Y., Ikeda, E., et al. (2011). Grafted human-induced pluripotent stem-cell-derived neurospheres promote motor functional recovery after spinal cord injury in mice. Proc. Natl. Acad. Sci. USA *108*, 16825–16830.

Nori, S., Okada, Y., Nishimura, S., Sasaki, T., Itakura, G., Kobayashi, Y., Renault-Mihara, F., Shimizu, A., Koya, I., Yoshida, R., et al. (2015). Long-term safety issues of iPSC-based cell therapy in a spinal cord injury model: oncogenic transformation with epithelial-mesenchymal transition. Stem Cell Rep. *4*, 360–373.

Ogura, A., Morizane, A., Nakajima, Y., Miyamoto, S., and Takahashi, J. (2013). gamma-secretase inhibitors prevent overgrowth of transplanted neural progenitors derived from human-induced pluripotent stem cells. Stem Cells Dev. *22*, 374–382.

Okano, H., and Yamanaka, S. (2014). iPS cell technologies: significance and applications to CNS regeneration and disease. Mol. Brain *7*, 22.

Okano, H., Nakamura, M., Yoshida, K., Okada, Y., Tsuji, O., Nori, S., Ikeda, E., Yamanaka, S., and Miura, K. (2013). Steps toward safe cell therapy using induced pluripotent stem cells. Circ. Res. *112*, 523–533.

Okubo, T., Iwanami, A., Kohyama, J., Itakura, G., Kawabata, S., Nishiyama, Y., Sugai, K., Ozaki, M., Iida, T., Matsubayashi, K., et al. (2016). Pretreatment with a gamma-secretase inhibitor prevents tumor-like overgrowth in human iPSC-derived transplants for spinal cord injury. Stem Cell Rep. *7*, 649–663.

Pfaff, N., Lachmann, N., Ackermann, M., Kohlscheen, S., Brendel, C., Maetzig, T., Niemann, H., Antoniou, M.N., Grez, M., Schambach, A., et al. (2013). A ubiquitous chromatin opening element prevents transgene silencing in pluripotent stem cells and their differentiated progeny. Stem Cells *31*, 488–499.

Pruszak, J., Ludwig, W., Blak, A., Alavian, K., and Isacson, O. (2009). CD15, CD24, and CD29 define a surface biomarker code for neural lineage differentiation of stem cells. Stem Cells *27*, 2928–2940.

Schuldiner, M., Itskovitz-Eldor, J., and Benvenisty, N. (2003). Selective ablation of human embryonic stem cells expressing a “suicide” gene. Stem Cells *21*, 257–265.

Seminatore, C., Polentes, J., Ellman, D., Kozubenko, N., Itier, V., Tine, S., Tritschler, L., Brenot, M., Guidou, E., Blondeau, J., et al. (2010). The postischemic environment differentially impacts teratoma or tumor formation after transplantation of human embryonic stem cell-derived neural progenitors. Stroke *41*, 153–159.

Sundberg, M., Bogetofte, H., Lawson, T., Jansson, J., Smith, G., Astradsson, A., Moore, M., Osborn, T., Cooper, O., Spealman, R., et al. (2013). Improved cell therapy protocols for Parkinson’s disease based on differentiation efficiency and safety of hESC-, hiPSC-, and non-human primate iPSC-derived dopaminergic neurons. Stem Cells *31*, 1548–1562.

Takahashi, K., Tanabe, K., Ohnuki, M., Narita, M., Ichisaka, T., Tomoda, K., and Yamanaka, S. (2007). Induction of pluripotent stem cells from adult human fibroblasts by defined factors. Cell *131*, 861–872.

Tateno, H., Onuma, Y., Ito, Y., Hiemori, K., Aiki, Y., Shimizu, M., Higuchi, K., Fukuda, M., Warashina, M., Honda, S., et al. (2014). A medium hyperglycosylated podocalyxin enables noninvasive and quantitative detection of tumorigenic human pluripotent stem cells. Sci. Rep. *4*, 4069.

Tateno, H., Onuma, Y., Ito, Y., Minoshima, F., Saito, S., Shimizu, M., Aiki, Y., Asashima, M., and Hirabayashi, J. (2015). Elimination of tumorigenic human pluripotent stem cells by a recombinant lectin-toxin fusion protein. Stem Cell Rep. *4*, 811–820.

Tohyama, S., Hattori, F., Sano, M., Hishiki, T., Nagahata, Y., Matsuura, T., Hashimoto, H., Suzuki, T., Yamashita, H., Satoh, Y., et al. (2013). Distinct metabolic flow enables large-scale purification of mouse and human pluripotent stem cell-derived cardiomyocytes. Cell Stem Cell *12*, 127–137.

Traversari, C., Marktel, S., Magnani, Z., Mangia, P., Russo, V., Ciceri, F., Bonini, C., and Bordignon, C. (2007). The potential immunogenicity of the TK suicide gene does not prevent full clinical benefit associated with the use of TK-transduced donor lymphocytes in HSCT for hematologic malignancies. Blood *109*, 4708–4715.

Uno, N., Uno, K., Komoto, S., Suzuki, T., Hiratsuka, M., Osaki, M., Kazuki, Y., and Oshimura, M. (2015). Development of a safeguard system using an episomal mammalian artificial chromosome for gene and cell therapy. Mol. Ther. Nucleic Acids *4*, e272.

Wu, C., Hong, S.G., Winkler, T., Spencer, D.M., Jares, A., Ichwan, B., Nicolae, A., Guo, V., Larochelle, A., and Dunbar, C.E. (2014). Development of an inducible caspase-9 safety switch for pluripotent stem cell-based therapies. Mol. Ther. Methods Clin. Dev. *1*, 14053.

Yanagida, A., Ito, K., Chikada, H., Nakauchi, H., and Kamiya, A. (2013). An in vitro expansion system for generation of human iPS cell-derived hepatic progenitor-like cells exhibiting a bipotent differentiation potential. PLoS One *8*, e67541.

Zhao, Q., Lu, B., George, S.K., Yoo, J.J., and Atala, A. (2012). Safeguarding pluripotent stem cells for cell therapy with a non-viral, non-integrating episomal suicide construct. Biomaterials *33*, 7261–7271.

Stem Cell Reports, Volume *8*

Supplemental Information

Fail-Safe System against Potential Tumorigenicity after Transplantation of iPSC Derivatives

Go Itakura, Soya Kawabata, Miki Ando, Yuichiro Nishiyama, Keiko Sugai, Masahiro Ozaki, Tsuyoshi Iida, Toshiki Ookubo, Kota Kojima, Rei Kashiwagi, Kaori Yasutake, Hiromitsu Nakauchi, Hiroyuki Miyoshi, Narihito Nagoshi, Jun Kohyama, Akio Iwanami, Morio Matsumoto, Masaya Nakamura, and Hideyuki Okano

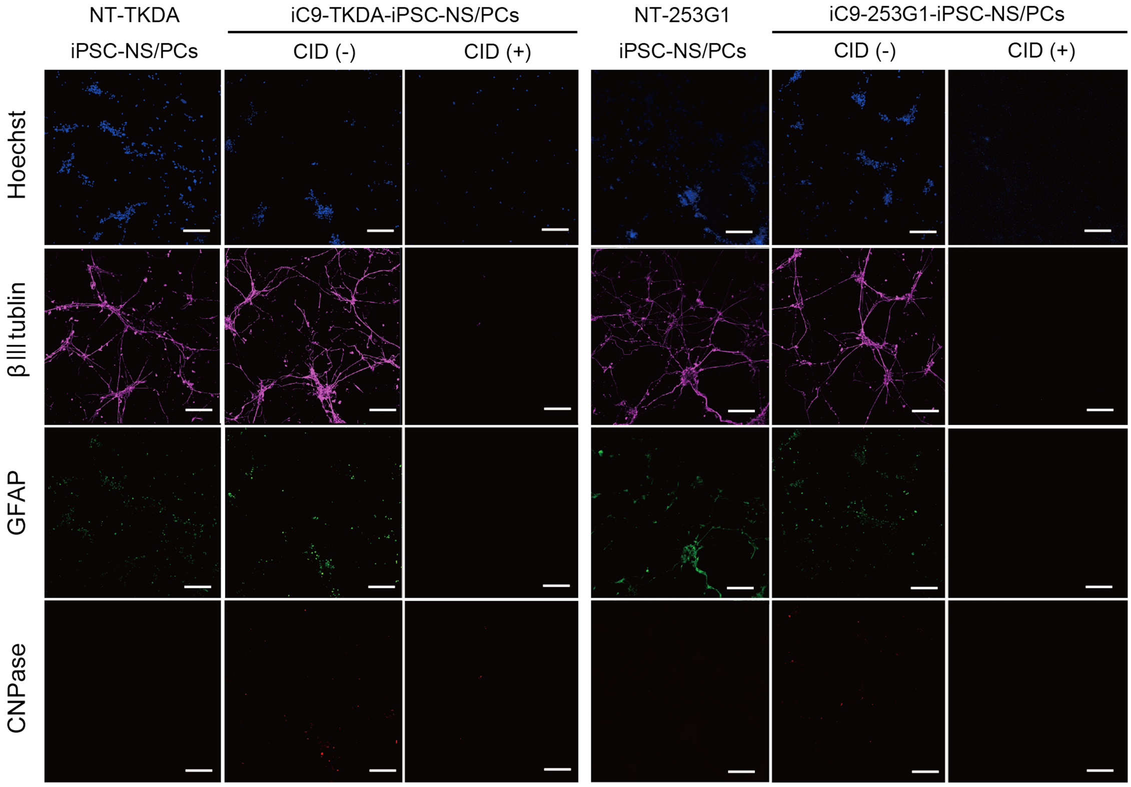

Figure S1. Integrated iC9 with CID-induced apoptosis in terminally differentiated iPSC derivatives

Terminally differentiated neurons and astrocytes from NT-iPSC-NS/PCs and iC9-iPSC-NS/PCs were treated with CID after 14 days of differentiation and stained. iC9-iPSC-NS/PCs differentiated into neurons and astrocytes similar to the NT-iC9-iPSC-NS/PCs. Activation of iC9 in these cells resulted in cellular apoptosis.

Scale bars = 100 μm.
